# Supplementary material for: A Multimodel Study of the Role of Novel PKC Isoforms in the DNA Integrity Checkpoint
Source: Int J Mol Sci. 2023 Oct 31;24(21):15796. doi: 10.3390/ijms242115796 (PMC10650207; doi:10.3390/ijms242115796)
Supplement: Supplementary file 1 [file ijms-24-15796-s001.zip › ijms-2663959-supplementary.pdf]

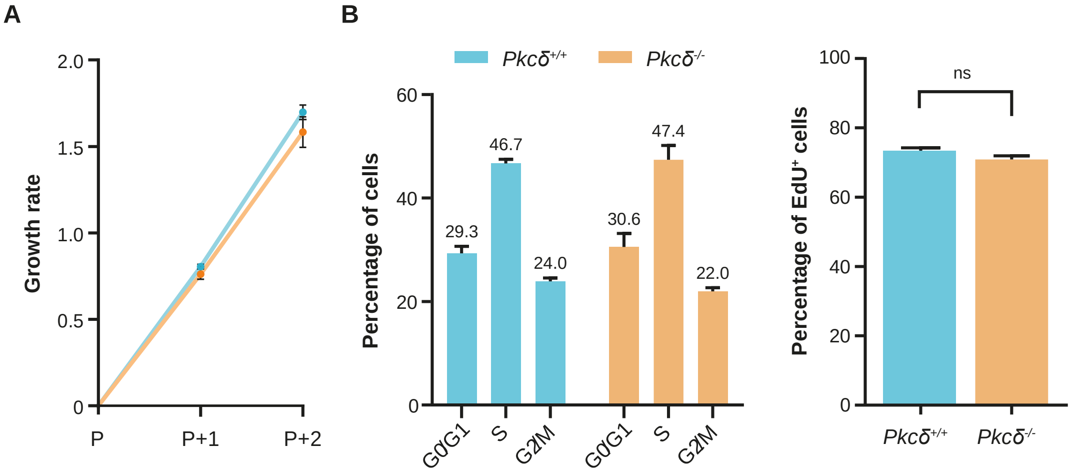

Figure S1. Analysis of cell growth and cell cycle dynamics of mESCs deficient for PKCδ. A) The growth of the *Pkcδ*$^{-/-}$ and *Pkcδ*$^{+/+}$ mESC lines was analyzed along two consecutive cell passages. To obtain the growth rate, the natural logarithm of the expansion factor (cell population divided by the number of cells initially plated) was in turn divided by the number of days. **B)** *Left panel:* Murine *Pkcδ*$^{-/-}$ and *Pkcδ*$^{+/+}$ mESCs were dissociated and stained with 25 µg/ml propidium iodide (Sigma Cat#P4864) with 25 µg/ml RNase (Roche Cat#10109142001) and their cell cycle distribution was analyzed using a BD FACSVerse flow cytometer. *Right panel*: Murine *Pkcδ*$^{-/-}$ and *Pkcδ*$^{+/+}$ ESCs received 10 µM EdU pulse for 1h before being dissociated and processed for EdU detection with the Click-iT^{TM} Plus EdU Alexa Fluor^{TM} 555 Flow Cytometry Assay Kit (ThermoFisher Cat#C10638). The samples were analyzed in a LSR-Fortessa flow cytometer to obtain the percentage of EdU^{+} cells.

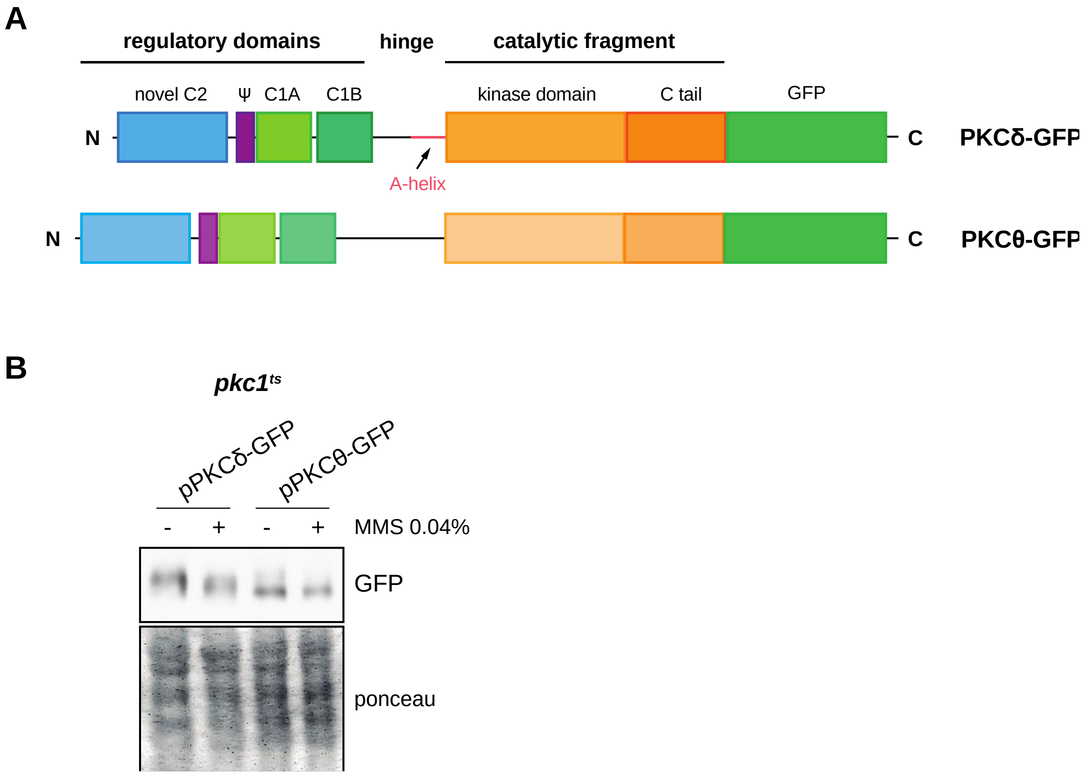

Figure S2. Protein level analysis of PKCδ and PKCθ. **A)** Schematic representation of GFP-tagged PKCδ and PKCθ proteins. **B)** Exponentially growing cultures of the *pkc1*^{ts} (JC6-3a) strain transformed with plasmids pPKCδ-GFP and pPKCθ-GFP were transferred to 37°C for 3 hours and then incubated in the absence or presence of MMS 0.04% for 1 hour. Protein levels of GFP-tagged PKCδ and PKCθ were detected by western blot. Ponceau staining of the membrane is shown as loading control.

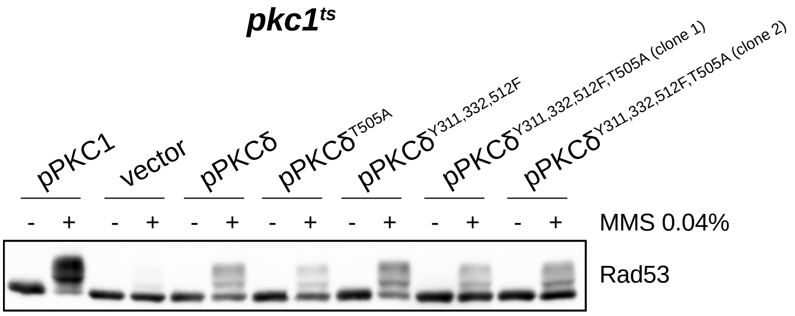

Figure S3. Analysis of DNA integrity checkpoint activation in key Tyr mutants in PKCδ oxidative stress response. Exponentially growing cultures of the *pkc1ts* (JC6-3a) strain transformed with plasmids pPKC1, pPKCδ, pPKCδ$^{Y311F, Y332F, Y512F}$, pPKCδ$^{Y311F, Y332F, Y512F, T505A}$ or with an empty vector were transferred to 37°C for 3 hours and then incubated in the absence or presence of MMS 0.04% for 1 hour. The activation of Rad53 was analyzed by western blot.

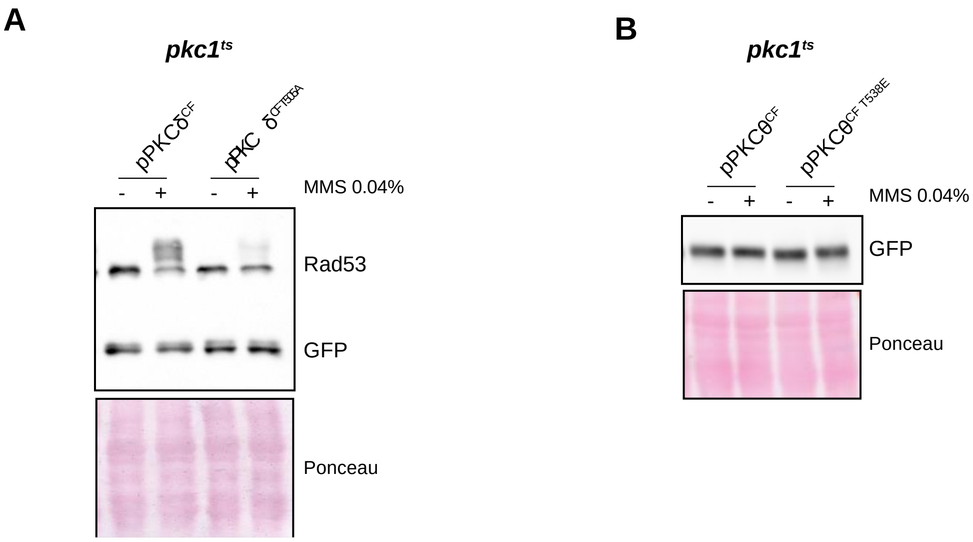

Figure S4. Protein level analysis of PKCδ^{CF} and PKCθ^{CF} mutants. Exponentially growing cultures of the *pkc1^{ts}* (JC6-3a) strain transformed with plasmids **A)** pPKCδ^{CF}-GFP and pPKCδ^{CFT505A}-GFP or **B)** pPKCθ^{CF}-GFP and pPKCθ^{CFT538E}-GFP were transferred to 37°C for 3 hours and then incubated in the absence or presence of MMS 0.04% for 1 hour. The activation of Rad53 and protein levels of GFP-tagged proteins were detected by western blot. Ponceau staining of the membrane is shown as loading control.

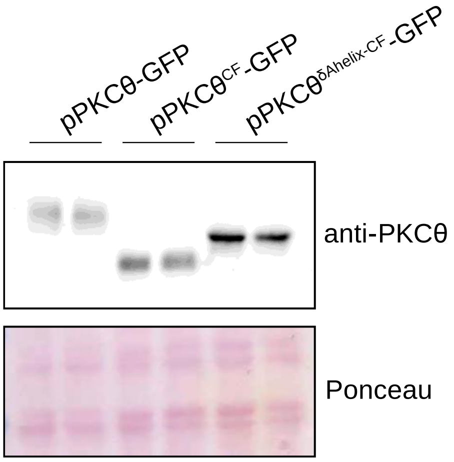

Figure S5. Protein level analysis of PKCθ truncated versions. Exponentially growing cultures of the *pkc1*^{ts} (JC6-3a) strain transformed with plasmids pPKCθ-GFP, pPKCθ^{CF}-GFP and pPKCθ^{CFT538E}-GFP were transferred to 37°C for 3 hours and then incubated in the absence or presence of MMS 0.04% for 1 hour. Protein levels were detected by western blot with a rabbit mAb anti-PKCθ antibody (Cell Signaling Cat#13643). Ponceau staining of the membrane is shown as loading control.

Table S1. Comparison of amino acids between novel PKCs. PKCδ residues involved in the A-helix dependent mechanism of activation loop stabilization and the corresponding residues of the PKCθ and PKCε isoforms.

nPKC	Contact 1		Contact 2		Contact 3	
**δ**	**Tyr^{332}**	**Phe^{498}**	**Trp^{336}**	**Arg^{397}**	**Ile^{497}**	**Phe^{525}**
**θ**	Gln	Leu	Trp	Arg	Met	His
**ε**	Asn	Met	Arg	Arg	Ile	Pro

Table S2. Mutant versions of PKCδ and PKCθ proteins.

Protein	Mutant version
**PKCδ**	$PKCδ^{T505A}$
	$PKCδ^{E500G}$
	$PKCδ^{T505A,E500G}$
	$PKCδ^{Y311,332,512F}$
	$PKCδ^{Y311,332,512F,T505A}$
**$PKCδ^{CF}$**	$PKCδ^{CF\ T505A}$
	$PKCδ^{CF\ E500G}$
	$PKCδ^{CF\ T505A,E500G}$
**$PKCδ^{Ahelix\text{-}CF}$**	$PKCδ^{Ahelix\text{-}CF\ T505A}$
	$PKCδ^{Ahelix\text{-}CF\ E500G}$
	$PKCδ^{Ahelix\text{-}CF\ T505A,E500G}$
	$PKCδ^{Ahelix\text{-}CF\ T505A,E500G,Y332Q,W336R,I497M,F498L,F525H}$
	$PKCδ^{Ahelix\text{-}CF\ T505A,I497A,F498A,F525A}$
**PKCθ**	$PKCθ^{T538A}$
**$PKCθ^{CF}$**	$PKCθ^{CF\ T538E}$

Table S3. Oligonucleotides used for site-directed mutagenesis. Mutated nucleotides are highlighted in bold.

Point mutation	Oligonucleotide	Sequence (5'-3')
**PKCδ Y311F**	forward	CTGTCGGAATAT**TT**CAGGGATTTGAGAAG
	reverse	CTTCTCAAATCCCTG**AA**ATATTCCGACAG
**PKCδ Y332F**	forward	CAACGGGACCT**T**TGGCAAGATCTGGG
	reverse	CCCAGATCTTGCCA**A**AGGTCCCGTTG
**PKCδ Y332Q**	forward	CTAGACAACAACGGGACC**CAA**GGCAAGATCTGGG
	reverse	CCCAGATCTTGCC**TTG**GGTCCCGTTGTTGTCTAG
**PKCδ W336R**	forward	GACCTATGGCAAGATC**C**GGGAGGGGAGCAC
	reverse	CGGGTGCTCCCCTCCC**G**GATCTTGCCATAG
**PKCδ I497M**	forward	CAAAGAGAATAT**G**TTTGGGGAG
	reverse	CTCCCCAAA**C**ATATTCTCTTTG
**PKCδ I497A**	forward	GTGCAAAGAGAAT**GCT**TTTGGGGAGG
	reverse	CCTCCCCAAA**AGC**ATTCTCTTTGCAC
**PKCδ F498L**	forward	GAATATATT**G**GGGGAGGGCCGG
	reverse	CCGGCCCTCCCC**C**AATATATTC
**PKCδ F498A**	forward	GTGCAAAGAGAATATA**GCT**GGGGAGGGC
	reverse	GCCCTCCCC**AGC**TATATTCTCTTTGCAC
**PKCδ E500G**	forward	GAATATATTTGGGG**GT**GGCCGGGCCAG
	reverse	CTGGCCCGGCC**AC**CCCCAAATATATTC
**PKCδ T505A**	forward	GGCCGGGCCAGC**GCT**TTCTGCGGC
	reverse	GCCGCAGAA**AGC**GCTGGCCCGGCC
**PKCδ Y512F**	forward	GGCACTCCTGACT**TT**ATCGCCCCTG
	reverse	CAGGGGCGAT**AA**AGTCAGGAGTGCC
**PKCδ F525H**	forward	GGCCTGAAGTACTCC**CAT**TCGGTGGACTG
	reverse	GTCCACCGA**ATG**GGAGTACTTCAG
**PKCδ F525A**	forward	GGCCTGAAGTACTCC**GCT**TCGGTGGACTG
	reverse	CAGTCCACCGA**AGC**GGAGTACTTCAGGCC
**PKCθ T538A**	forward	GCGAAGACAAAT**G**CTTTCTGTG
	reverse	CACAGAAAG**C**ATTTGTCTTCGC
**PKCθ T538E**	forward	GAAGACAAAT**GAA**TTCTGTGGAACTC
	reverse	GAGTTCCACAGAA**TTC**ATTTGTCTTC
